# A Case of Diastematomyelia Presenting With Minimal Neurologic Deficits in a Middle-Aged Patient

**DOI:** 10.7759/cureus.12621

**Published:** 2021-01-11

**Authors:** Gabriella Mamo, Rishu Batra, Jeffrey Steinig

**Affiliations:** 1 Radiology, Philadelphia College of Osteopathic Medicine, Philadelphia, USA; 2 Neuroradiology, Roxborough Memorial Hospital, Philadelphia, USA

**Keywords:** diastematomyelia, congenital, spine, spinal cord, dysraphism, anomaly, vertebra, neurology

## Abstract

Diastematomyelia is a rare congenital deformity of the spine in which the spinal cord is split into two hemicords along the sagittal plane. This condition belongs to the group of spinal dysraphisms, is more common in females, and is usually diagnosed prenatally or during childhood; rarely is it diagnosed in adults. We report a male patient in his 50s in which diastematomyelia of the thoracic spine was incidentally encountered after receiving a CT scan of the chest for shortness of breath. Although most patients with this condition are symptomatic, the patient did not display any significant acute neurological complaints at the time. The patient had a history of spina bifida and is paraplegic, both of which are commonly associated with diastematomyelia. The lack of progressive neurologic symptoms, diagnosis in the patient’s adult life, and the presence of the anomaly solely in the thoracic spine make this a rare and unusual case. Early recognition and diagnosis of this condition, by prenatal ultrasound or MRI, can help to prevent further damage to the spinal cord and allow affected patients to seek treatment sooner, thus improving quality of life.

## Introduction

Diastematomyelia is a rare congenital anomaly characterized by a longitudinal splitting of the spinal cord into two hemicords. This disorder belongs to the group of spinal dysraphisms, along with other disorders such as meningocele, myelomeningocele, and spina bifida occulta. It also often coexists with these spinal dysraphisms in addition to others, or vertebral anomalies such as scoliosis, hemivertebrae, or butterfly vertebrae [1-3].

In this abnormality, each hemicord includes a central canal, dorsal root, and ventral root. It is believed that diastematomyelia is caused by the presence of a bony, fibrous, or cartilaginous septum that moves dorsally towards the vertebral canal, arising from the posterior surface of the anterior wall during development. This leads to abnormal development of the notochord during the 15-18th days of pregnancy [1-3]. MRI is the diagnostic test of choice for this disorder [4].

Diastematomyelia is divided into two types. Type I, the classic type, is characterized by two dural sacs enclosing each hemicord with a common septum or spur (bony, cartilaginous, or fibrous) dividing the spinal canal. Patients with this type are usually symptomatic. Type II is characterized by a single dural sac surrounding both hemicords, lacking a septum in most cases. Patients with Type II are typically asymptomatic [1].

This anomaly is typically diagnosed in childhood or during the prenatal period and is more common in females (80-90%); initial diagnosis is rarely established in adulthood. Most cases occur in the thoracolumbar areas and are rarely restricted to the thoracic or lumbar spine exclusively. As described in the literature, diastematomyelia is often associated with various orthopedic and dermatologic abnormalities such as congenital scoliosis, clubfoot, lower limb discrepancy, hairy patches, dimples, lipomas, sinus tracts, or hemangiomas [5, 6]. The majority of patients with this disorder are symptomatic. The clinical presentation of diastematomyelia is similar to that of tethered cord syndrome, in that most patients present with neurologic disturbances such as back pain, asymmetric reflexes, progressive weakness, muscle atrophy, loss of sensation, paresthesias, bowel and bladder dysfunction, spasticity, or paresis. The reason for these deficits is likely due to damage and tension on the spinal cord due to limited space in the vertebral canal [1, 2].

## Case presentation

**Figure 1:**
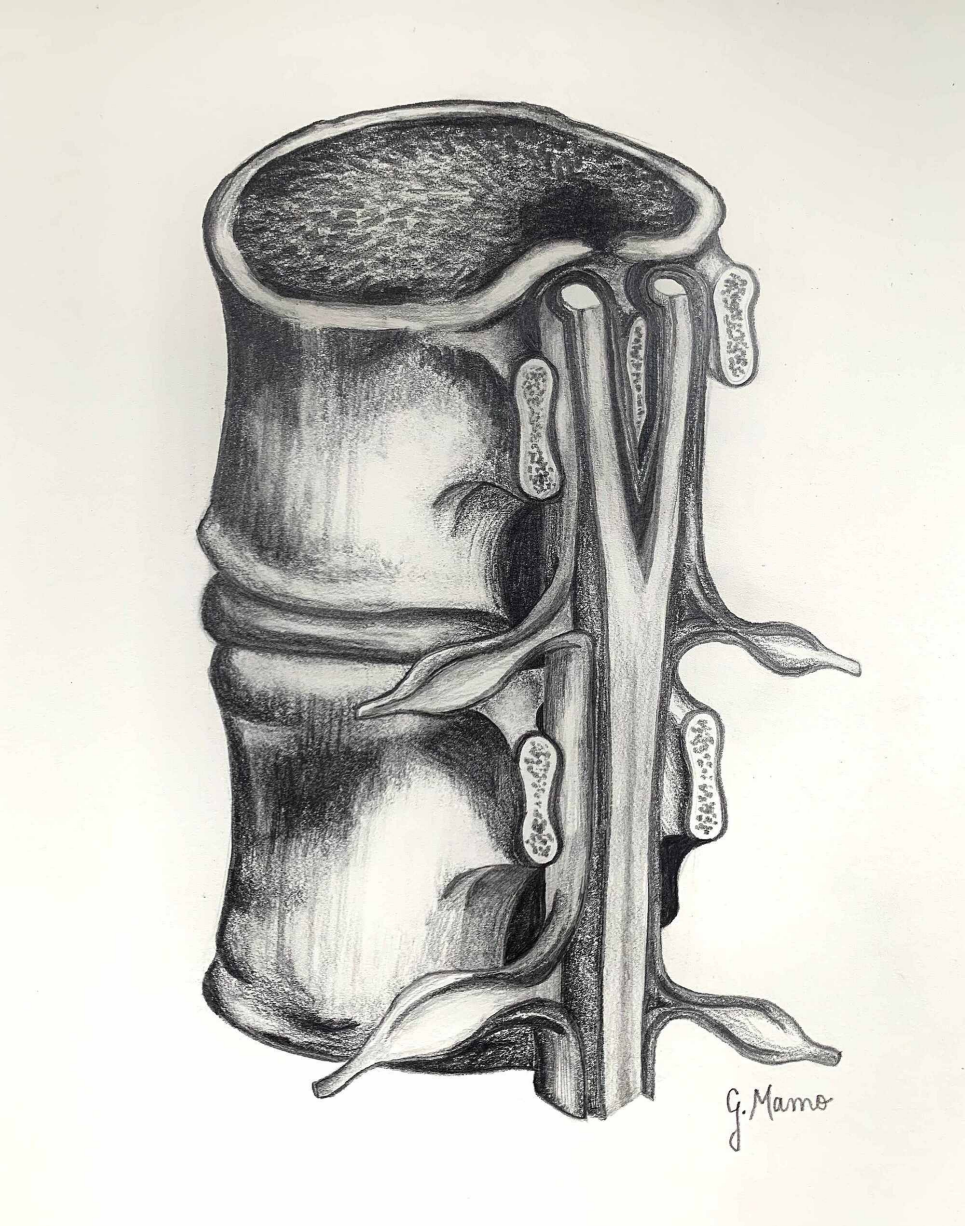
Diastematomyelia. (Figure illustration created by Gabriella Mamo.)

**Figure 2:**
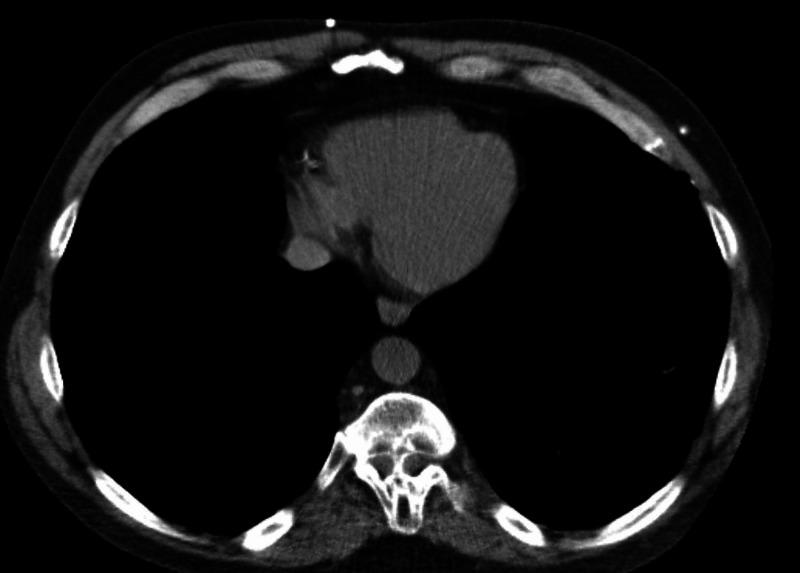
CT of the chest without contrast, axial views. This image demonstrates diastematomyelia of the thoracic spine with a bony septum at approximately T9-T11. There are also partially bifid vertebral bodies at T10 and T11.

**Figure 3:**
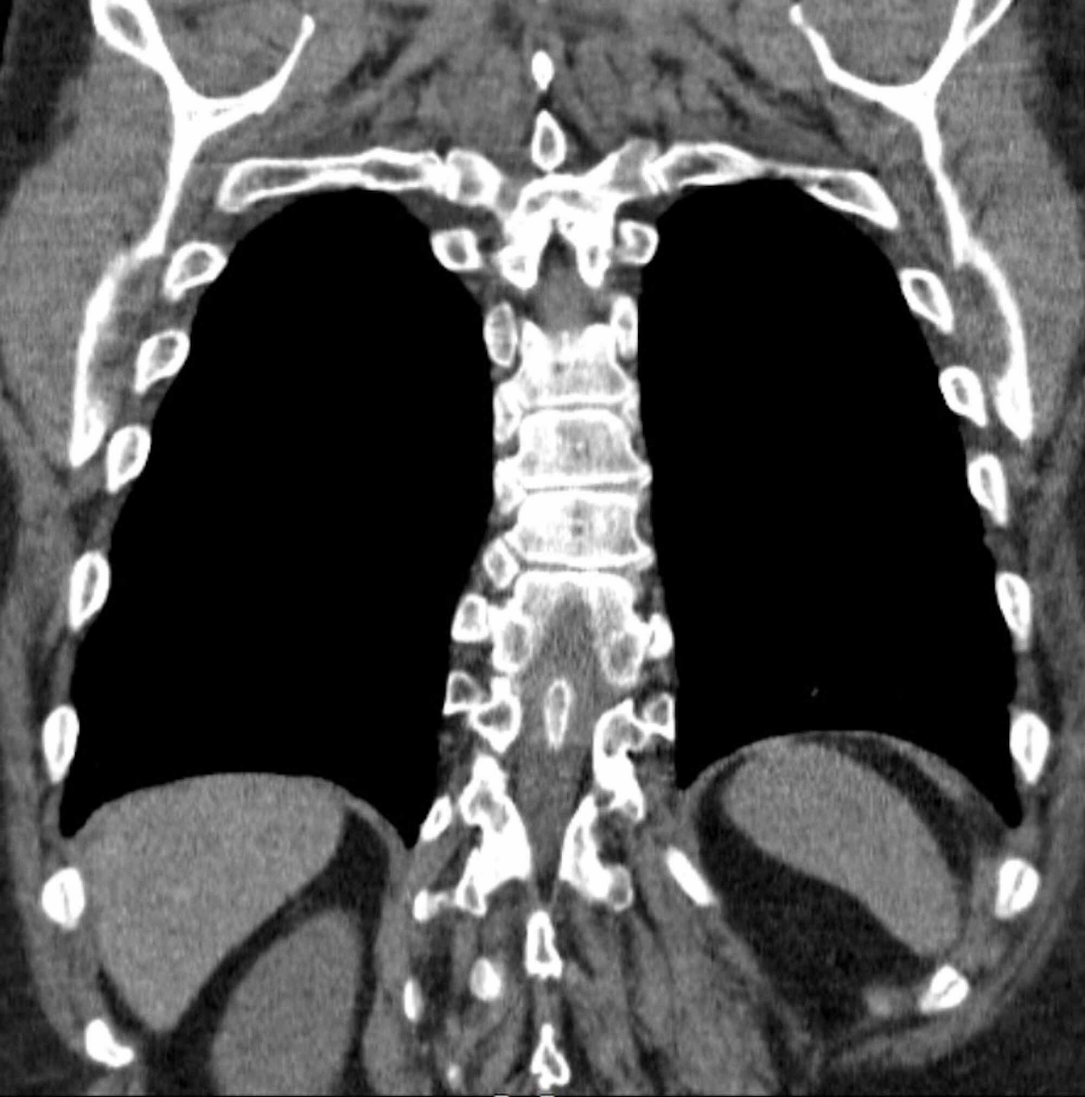
CT of the chest without contrast, coronal view. This shows an osseous structure dividing the vertebral canal in two as well as mild thoracic levoscoliosis.

**Figure 4:**
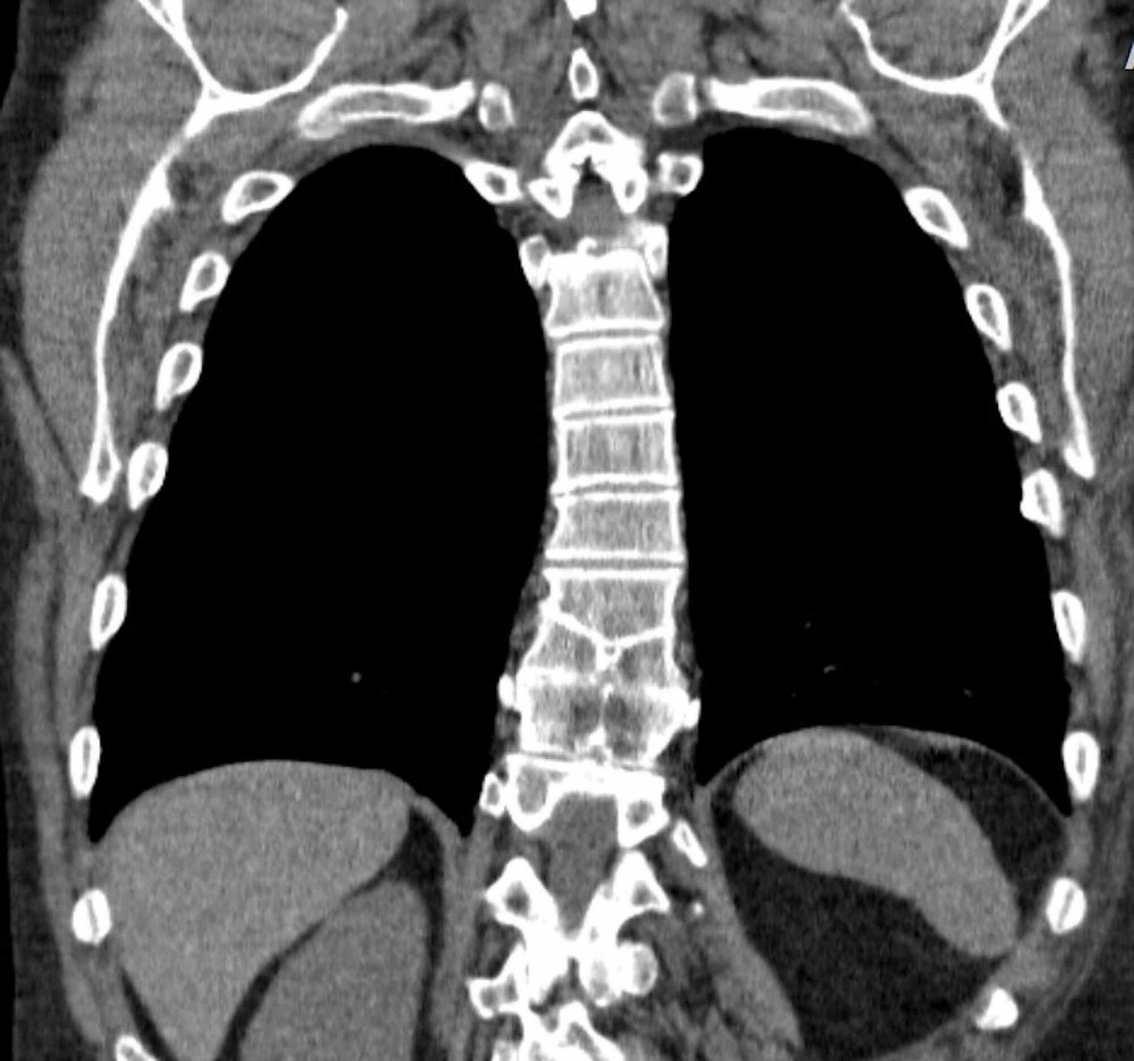
CT of the chest without contrast, coronal view. This view demonstrates a T10 butterfly vertebra as well as fusion of T9-T10 and T10-T11.

We report a male patient in his 50's with a past medical history of chronic obstructive pulmonary disease (COPD), spina bifida of the lumbar region with residual paraplegia, and osteoarthritis who presented as an outpatient for radiologic imaging. He was referred by his pulmonologist to obtain a non-contrast CT scan of the chest due to shortness of breath. In addition to the lung findings, a bony septum separating two hemicords in the vertebral canal was noted, consistent with diastematomyelia of the thoracic spine (Figures 1-4). 

This incidental spinal abnormality has been previously documented a few years prior in a non-contrast CT of the chest, again ordered for his lung disorders. The patient is wheelchair-bound, and his family history includes spina bifida in his biological sister. A physical exam from a few months earlier showed 5/5 muscle strength in the bilateral upper extremities without pronator drift and paraplegia in the bilateral lower extremities. Sensation to light and sharp touch was intact in all four extremities. There were 2+ reflexes throughout, and Hoffmann sign absent bilaterally. Previous medical records also indicated that the spine was nontender to palpation with inferior dimpling, as well as the presence of contractures of the feet and lower extremities. Interestingly, from the time of the diagnosis to the present, the patient did not have any significant progressive or acute neurological complaints related to diastematomyelia. 

## Discussion

There are several imaging modalities used to make the diagnosis of diastematomyelia. Plain radiographs may show evidence of bony malformations, scoliosis, and spina bifida, but these abnormalities, in addition to others, are able to be more clearly appreciated on CT scan. However, MRI is the most ideal diagnostic tool for assessing spinal dysraphisms, as it can better elucidate a split spinal cord and the presence of other associated abnormalities [7]. 

Imaging features of diastematomyelia may correlate with the clinical manifestations of the lesion. A neurologic grading system can be used to establish a clinical diagnosis. This includes gait, sphincter function, motor function, tendon reflex and sensation. Findings on combined CT and MRI studies, including symmetry of splitting, presence of septum, and location of the lesion, will help predict the clinical implications of this lesion. This is important in helping guide management and future prognosis [4].

Early diagnosis of this anomaly could allow for earlier surgical intervention and thus yield a better prognosis compared to a diagnosis later in adult life. Prenatal ultrasound is an ideal method of diagnosis of diastematomyelia, along with other spinal dysraphisms, and is usually able to be detected during the third trimester. The fetal ultrasound would show an additional midline posterior echogenic focus in the spine between the laminae [5]. One would also see hyperechogenic masses usually located behind the ossification center of vertebral bodies. The usual division of the spinal cord can be seen in the transverse plane [8].

As discussed above, this patient presented in his adult life having almost no acute or progressive neurological complaints or findings on physical exams related to diastematomyelia with the exception of his paraplegia. Not only is this very uncommon, but the patient’s diastematomyelia was also found exclusively within his thoracic spine. This suggests that not only the lumbar spine, but also the thoracic spine, should be evaluated when screening for diastematomyelia. The patient also had spina bifida, scoliosis, vertebral fusion, and butterfly vertebrae, all of which are common in patients with the anomaly. The family history of spina bifida in the patient’s biological sister may suggest genetic or environmental causes, such as inadequate intake of folic acid during the mother’s pregnancy. As a result, early screening and diagnosis, as well as adequate prenatal care, are essential for prevention and treatment of this disorder.

Treatment for diastematomyelia is indicated if progressive neurological symptoms are present. One study notes that surgical resection of the spur should be performed if the patient has worsening neurological symptoms; otherwise, patients who are asymptomatic or do not have neurological deterioration should be observed [9]. In addition, if patients show signs and symptoms consistent with tethered cord, surgery should also be performed. Early surgical treatment may be able to prevent further damage to the spinal cord.

## Conclusions

Diastematomyelia is a rare congenital defect of the spinal cord belonging to the group of spinal dysraphisms. This condition is more common in females and is rarely diagnosed in adulthood. It is commonly found with other spinal abnormalities, such as scoliosis, butterfly vertebrae, vertebral fusion, hemivertebrae, or spina bifida. Imaging plays an important role in the diagnosis of diastematomyelia, especially because some patients may not present with any acute neurologic symptoms, as was our reported patient. However, neurological deterioration may eventually occur due to damage to the spinal cord; as a result, it is essential to establish an early diagnosis in order to improve patients’ quality of life. If prenatal ultrasound or other imaging modalities are unable to be performed, it is important to be aware of other dermatological manifestations that spinal dysraphisms can cause, such as dorsal hair patches, dimples, lipomas, and hemangiomas. Awareness of these anomalies can aid in the recognition and diagnosis of a spinal dysraphism, and thus expedite appropriate treatment.

## References

[1] Zaleska-Dorobisz U, Bladowska J, Biel A, Pałka LW, Hołownia D (2010). MRI diagnosis of diastematomyelia in a 78-year-old woman: case report and literature review. Pol J Radiol.

[2] Vissarionov SV, Krutelev NA, Snischuk VP (2018). Diagnosis and treatment of diastematomyelia in children: a perspective cohort study. Spinal Cord Ser Cases.

[3] Neuhauser EB, Wittenborge MH, Dehlinger K (1950). Diastematomyelia; transfixation of the cord or cauda equina with congenital anomalies of the spine. Radiology.

[4] Huang SL, He XJ, Xiang L, Yuan GL, Ning N, Lan BS (2014). CT and MRI features of patients with diastematomyelia. Spinal cord.

[5] Azimi P, Mohammadi HR (2013). Diastematomyelia presenting with no pain in a 53-year-old man: a case report. Iran Red Crescent Med J.

[6] Bekki H, Morishita Y, Kawano O, Shiba K, Iwamoto Y (2015). Diastematomyelia: a surgical case with long-term follow-up. Asian Spine J.

[7] Kachewar SG, Sankaye SB (2014). Diastematomyelia - a report of two cases. J Clin Diagn Res.

[8] Wei Q, Cai A, Wang X, Wang X, Xie L (2017). The value of prenatal ultrasonographic diagnosis of diastematomyelia. J Ultrasound Med.

[9] Miller A, Guille JT, Bowen JR (1993). Evaluation and treatment of diastematomyelia.. JBJS.

